# Nano- and Microdelivery Systems for Marine Bioactive Lipids

**DOI:** 10.3390/md12126014

**Published:** 2014-12-17

**Authors:** David M. Pereira, Patrícia Valentão, Paula B. Andrade

**Affiliations:** REQUIMTE/Laboratory of Pharmacognosy, Department of Chemistry, Faculty of Pharmacy, University of Porto, Rua de Jorge Viterbo Ferreira n° 228, 4050-313 Porto, Portugal; E-Mails: dpereira@ff.up.pt (D.M.P.); valentao@ff.up.pt (P.V.)

**Keywords:** fatty acids, ω-3, liposomes, microparticles, nanoparticles, EPA, DHA

## Abstract

There is an increasing body of evidence of the positive impact of several marine lipids on human health. These compounds, which include ω-3 polyunsaturated fatty acids, have been shown to improve blood lipid profiles and exert anti-inflammatory and cardioprotective effects. The high instability of these compounds to oxidative deterioration and their hydrophobicity have a drastic impact in their pharmacokinetics. Thus, the bioavailability of these compounds may be affected, resulting in their inability to reach the target sites at effective concentrations. In this regard; micro/nanoparticles can offer a wide range of solutions that can prevent the degradation of targeted molecules, increase their absorption, uptake and bioavailability. In this work we will present the options currently available concerning micro- and nanodelivery systems for marine lipids; with emphasis on micro/nanoparticles; such as micro/nanocapsules and emulsions. A wide range of bottom-up approaches using casein, chitosan, cyclodextrins, among others; will be discussed.

## 1. Fatty Acids in Health & Disease

Fatty acids are lipophilic molecules that can be found in all living organisms. They can occur in their free form or, alternatively, integrate more complex lipids, such as triglycerides, phospholipids or glycolipids. In addition, fatty acids can be part of lipoproteins, lipopolysaccharides and alkaloids, among others.

As it has been reviewed before [1], fatty acids exhibit a very diversified chemistry, which results in several possible structures that can be saturated/unsaturated, branched or cyclic, and with distinct functional groups, such as hydroxyl, keto and epoxy in some taxonomic groups.

Lipids in general, fatty acids in particular, serve three major roles in organisms: they are structural components of biological membranes, provide energy reserves and serve as biologically active molecules exerting a wide range of functions. Several epidemiological studies, as well as randomized and controlled trials, show that polyunsaturated fatty acids (PUFA), such as eicosapentaenoic acid (EPA) and docosahexaenoic acid (DHA), have benefic effects on cardiovascular diseases by lowering cardiovascular risk [2,3,4,5,6]. The mechanisms by which this effect is accomplished are diverse and include lowering of blood lipid levels, improved endothelial function and attenuated inflammatory responses [5,7].

Many inflammatory conditions result from an excessive production of pro-inflammatory mediators like eicosanoids, prostaglandin E_2_ (PGE_2_) and leukotriene B4 (LTB_4_). These molecules are synthesized in the organism from the ω-6 FA arachidonic acid (C20:4 ω-6). If we consider that western diet results in high ω-6 and low ω-3 ratio of PUFA [8], it may be postulated that correction of these proportions, by increasing the consumption of ω-3 FA like EPA (C20:5 ω-3) and DHA (C22:6 ω-3), may change this trend [9], as they may replace arachidonic acid as an eicosanoid substrate in cell membranes [10]. In addition, several works have shown that the anti-inflammatory properties of FA can also occur downstream of this process [11].

Regardless of the diversity of their biological effects, one of the factors that hinder the use of fatty acids in health and disease is related to their bioavailability.

In this regard, nano- and microsystems constitute important strategies for the delivery of these molecules, by providing both increased dissolution and protection against degradation [12,13,14,15,16,17,18,19,20].

## 2. Nano- and Microencapsulation

The general concept of encapsulation refers to a process by which a material (solid, liquid or gas) is packed within sphere-shaped structures. According to the particle size, microparticles or nanoparticles can be obtained, their applications being found across several areas, e.g., in food and pharmaceutical industries [21]. Particles in the 1–800 μm range are known as microparticles, microspheres or microcapsules, while others below 1 μm are considered to be nanoparticles, nanospheres or nanocapsules [22], although some authors prefer to refer to nanoparticles only when their size is lower than 100 nm.

Microencapsulation technology has initially been used almost exclusively in the pharmaceutical industry as a method for protecting sensitive components and, sometimes, as a way to increase drug delivery [23,24,25]. Given the potential of this approach, it has been extended to other industries, namely the food industry, as a strategy for protecting nutrients and delaying oxidation [26,27]. Microencapsulation is a process in which small particles of the active and/or sensitive component, such as fish oil or pure fatty acids, known as the core, are packaged within an encapsulating matrix [28].

Nanoparticles can be prepared by two basic approaches: either by a ‘‘top-down’’ approach, in which nanoparticles are produced by means of physical processing of several materials, or alternatively by a ‘‘bottom-up’’ approach, in which nanoparticles are produced *via* self-assembly and self-organization of smaller molecules [1]. Nowadays the general approach relies in both strategies to produce bioactive molecules-loaded nanoparticles that will result in improved availability in the human body. Thus, the efficacies of active ingredients, such as phytochemicals, vitamins, nutrients, or minerals, are preserved against the destructive conditions that oral consumption and absorption through the digestive track represent [13].

The differences between nano- and microparticles extend beyond the size, translating into their chemical and physical characteristics, which, in turn, have a marked impact in their application and potential use. An interesting example is that of microemulsions *vs* nanoemulsions.

Nanoemulsions are droplets of multiphase colloidal dispersions that are obtained from one liquid in another immiscible liquid, the chemical identity of the components and techniques used playing a pivotal role in the size of the droplets [29,30,31,32].

Several characteristics distinguish nanoemulsions from their micro- counterparts. From an optical point of view, nanoemulsions are frequently transparent owing to their size, which is smaller than visible wavelengths. Differently, microemulsions scatter visible light, thus originating a white opaque appearance [30]. Another interesting feature of nanoemulsions relies in their metastability, thus allowing them to be diluted with water without relevant changes in the distribution of droplet size [33].

In the specific case of marine ω-3 fatty acids, one common drawback of their use and formulation is their hydrophobicity and the rate at which they oxidize, thus originating secondary lipids oxidation products that arise from the decomposition of hydroperoxides, mainly aldehydes, ketones and alcohols of distinct chain lengths and degrees of unsaturation, such as (*Z*)-1,5-octadiene-3-one, (*E*,*Z*)-2,4-heptadienal, 1-penten-3-ol and (*Z*)-4-heptenal [16,34]. Most of these compounds display low odor thresholds, thus affecting the sensory quality at very low concentrations [16,35]. For this reason, stabilization in aqueous medium and protection against *stimuli* that trigger deterioration is required. In the case of ω-3 fatty acids and other components of fish oil, micro/nanoencapsulation is successfully used to protect against photo-oxidation and appearance of oxidized off-flavors, thus contributing to their quality and increased shelf-life [14,15,16,36]. By controlling the properties of the materials used and variables like pH and temperature, controlled release of the core materials can be achieved. In what concerns to stability, several factors besides composition are known to interfere, namely drying methods like spray granulation, spray drying and freeze drying [36].

From an experimental point of view, the rate of oxidation and formation of secondary lipids oxidation products is frequently evaluated by measuring lipid hydroperoxides, thiobarbituric acid reactive substances (TBARS) and headspace propanal/hexanal by gas chromatography- solid phase microextraction (GC-SPME).

## 3. Case Studies: Micro- and Nanodelivery Systems for Fatty Acids

### 3.1. Lipidic Systems

#### 3.1.1. Liposomes

**Figure 1:**
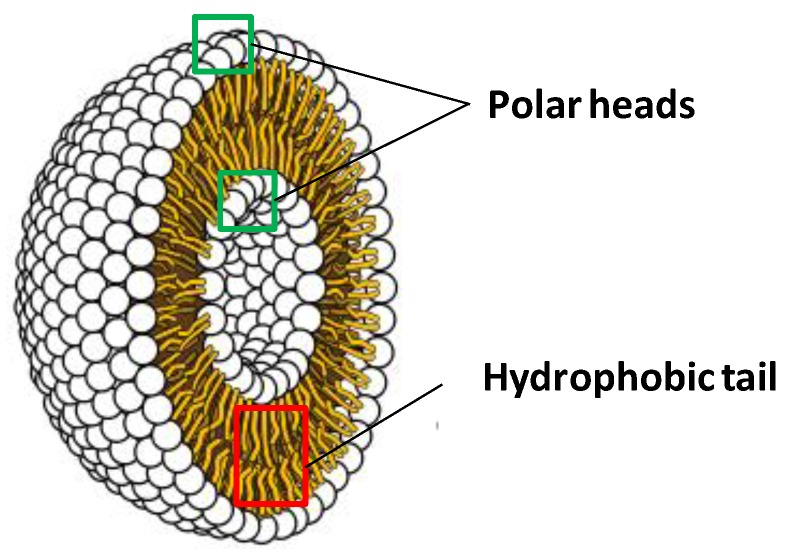
General structure of a liposome.

Liposomes are bilayer spherical structures constituted by surfactants, such as phospholipids (Figure 1). They are obtained by self-assembly of lipid molecules in aqueous systems, owing to their amphiphilic molecules, which adopt the bilayer sphere architecture as a way to shield the hydrophobic groups in their interior while maintaining the polar head groups in contact with the aqueous phase [37,38].

Owing to their chemical robustness, liposomes can be used to formulate, carry, deliver and release water-soluble, lipid-soluble and amphiphilic molecules [39,40,41,42].

From a physical point of view, liposomes can present sizes ranging from 20 nm to a few hundred µm [43]. Several factors, including stability, are markedly affected by intrinsic and extrinsic factors. As intrinsic factors we can refer the nature and identity of the liposome components and trapped molecules which, in turn, affect both the rheological properties and surface charge [44,45]. In the case of surface charge, its impact in electrostatic interactions is of pivotal importance in designing liposomes, due to its relevance in pharmacokinetics, for instance by affecting disintegration and hence the release profile of trapped compounds. Likewise, extrinsic factors, such as temperature, pH and ionic strength, have considerable influence in the chemical and physical stability of liposomes [46].

Temperature-sensitive liposomes can be produced by modifying the lipid layers with specific polymers that changes their solubility at a critical temperature, thus destabilizing the liposome membrane [30]. However, to the best of our knowledge, this approach has yet to be applied to liposomes with marine fatty acids.

In an interesting work, Cansell *et al*., used liposomes produced from an extract of marine lipids rich in ω-3 PUFA as a delivery system for fatty acids supplements, thus increasing their bioavailability in rats [47]. The lipid profile of the animals was compared after feeding with regular fish oil and fish oil incorporated in the liposomes membranes. When using liposomes, fatty acid absorption was 98% ± 1%, while in the case of plain fish oil this value was reduced to 73% ± 6%. When studying individual compounds, DHA proportion in lymph was higher after liposome ingestion (78%) than after fish oil ingestion (47%). Interestingly, phospholipids concentration in lymph was not affected, regardless of the fat source, which is compatible with phospholipid regulation by *de novo* triacylglycerol (TAG) synthesis. An interesting result was that the distribution of *n*-3 PUFA esterified on the *sn-2* position of chylomicron TAG depended on the lipid source administered.

This *in vivo* study confirmed that marine lipids are an interesting strategy for the delivery of PUFAs to the organism [47].

#### 3.1.2. Multi-Layered Emulsions

Multi-layered emulsions constitute a robust strategy for protecting fish oils against oxidation. This is achieved by controlling each layer’s composition, net electrical charge, permeability, and environmental responsiveness which, in turn, allows minimizing the exposure to temperature, mechanical agitation and pH [13].

Monodispersed fish oil-in-water emulsions have been laminated by applying several interfacial membranes in a layer-by-layer (LbL) approach [48]. While the primary emulsion was obtained through a membrane homogenizer using anionic citric acid ester of mono- and diglyceride, the second and tertiary emulsions relied on the electrostatic deposition of cationic chitosan and anionic sodium alginate, respectively, on the surfaces of the oil droplets. The positively charged secondary emulsions (+56.27 ± 2.5 mV) were more stable to lipid oxidation when compared to negatively charged primary (−45.13 ± 1.7 mV) and tertiary emulsions (−24.8 ± 1.2 mV), as evaluated by the levels of hydroperoxides, TBARS and headspace propanal/hexanal by GC-SPME. When evaluating the rate of digestion of oil droplets as a function of the different layers, lipid digestion was found to decrease with multilayer coating [48].

In another work, multilayer emulsifier systems comprising β-lactoglobulin and citrus/sugar beet pectin were tested [13]. Sugar beet pectin was used as it contains ferulic acid, thus being an antioxidant candidate for preventing oxidation of fish oil. Oil-in-water emulsions with β-lactoglobulin were prepared, upon which pectins were electrostatically deposited. Emulsions prepared with 1% oil, 0.05% β-lactoglobulin and 0.06% pectins were physically stable for up to 16 days. Emulsions prepared with the multilayer system of β-lactoglobulin and citrus pectin were more stable than emulsions stabilized with β-lactoglobulin alone, as revealed by monitoring lipid hydroperoxide-derived propanal formation. Emulsions prepared with β-lactoglobulin plus sugar beet pectin were less stable than emulsions stabilized with β-lactoglobulin alone, despite the presence of ferulic acid in the sugar beet pectin [13].

The authors hypothesized that the presence of high levels of iron and copper could result in oxidative stress that surpassed the antioxidant capacity of ferulic acid [13].

In another study using a LbL approach (from menhaden oil) the primary layer was lecithin and the secondary one was chitosan-lecithin, microencapsulation being achieved by spray-drying with corn syrup [49]. In this system the interfacial properties of microencapsulated lecithin-chitosan multilayer emulsion droplets remained intact upon reconstitution into an aqueous system, the reconstituted secondary emulsion being more stable than the primary one. More physically stable powders were obtained when high amounts of corn syrup (10%–20%) were used [49].

#### 3.1.3. Cochleates

Cochleates are a lipid-based system obtained from the precipitation of negatively charged lipids and cations [50].

**Figure 2:**
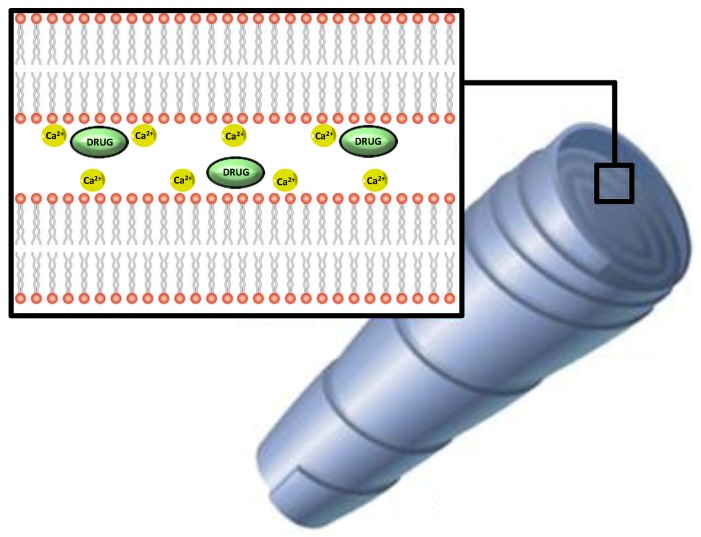
Schematic representation of nanocochleates.

One of the most widespread production processes is the crystallization of phospholipids from soy in the presence of calcium. From a structural point of view, these particles comprise phospholipid bilayers stacked as sheets and rolled in a spiral configuration, with solutions of multivalent cations locating between each sheet, “wrapping” around oil droplets containing the molecules to be trapped (Figure 2).

Experimentally, these particles can be obtained by slow introduction of cations into suspensions of anionic liposomes, thus causing the liposomes to fuse together, cochleate formation being indicated by a rapid increase of turbidity [51]. As reviewed by Loveday *et al.*, small unilamellar liposomes prepared by film hydration give more uniform cigar-shaped cochleates than multilamellar liposomes from powdered phospholipids [52]. Differently, in the hydrogel-based method, liposomes are mixed with a polymer and injected into a solution of a second, non-miscible polymer, e.g., dextran and polyethylene glycol (PEG). Calcium is added to the water-in-water emulsion and diffuses slowly from the PEG continuous phase into the dispersed dextran-liposome phase, producing nanocochleates [53]. Sub-micrometre cochleates can be produced by this method, whereas the trapping method gives larger cochleates.

At least one product using this technology is currently being marketed as a tool for protecting ω-3 fatty acids, with claims upon enhanced stability (BioGeode™, by BioDelivery Sciences International, Inc., Raleigh, NC, USA).

#### 3.1.4. Simple Emulsion-Based Systems

Fish oil was has been microencapsulated using a matrix consisting of sugar beet pectin and glucose syrup [54]. Several experimental conditions were evaluated and median oil droplet size was shown to be markedly affected by the composition of the emulsion, as well as by the homogenization pressure. The best results were obtained when the emulsion was produced with 50% oil and around 2% of sugar beet pectin, yielding droplet size below 2 μm and maximum viscosity of 179 mPa [54].

An encapsulation technique for tuna oil using an ultrasonic atomizer and a process workflow comprising three steps (emulsification, ultrasonic atomization and freeze drying) has been reported [55]. Distinct wall materials, such as chitosan, maltodextrin and whey protein isolate were tested, together with their respective proportions. Chitosan in combination with maltodextrin yielded the particles with the smallest size and the highest emulsion stability. Gas chromatography-flame ionization detection (GC-FID) studies have assessed the effect of encapsulation on the fatty acids profile of fish oil. Prior to encapsulation, saturated fatty acids and PUFA were major compounds. Encapsulation led to EPA and DHA levels of 24 g/100 g, with simultaneous increase of the levels of the monounsaturated fatty acid (MUFA) eicosenoic acid [55]. EPA and DHA content of the encapsulated fish oil was higher than the commercial specification (10 g/100 g) [55].

Nanocapsules of α-linolenic acid have been produced through a modified emulsion diffusion technique [56]. Several conditions were tested, namely the use of polylactic acid as the encapsulating polymer, acetone and ethyl acetate as organic solvents and also tween 20, gelatin and Pluronic-F68 as stabilizers. When comparing all conditions, acetone revealed to be better than ethyl acetate, while Tween 20 exceeded Pluronic-F68. For the formation of smaller nanocapsules, an organic to aqueous phase ratio of 1:5 was the most suitable. Parameters, such as particle size and zeta potential, were monitored, values in the range of 100 nm and +33 mV being obtained [56].

### 3.2. Non-Lipidic Systems

#### 3.2.1. Cyclodextrins

**Figure 3:**
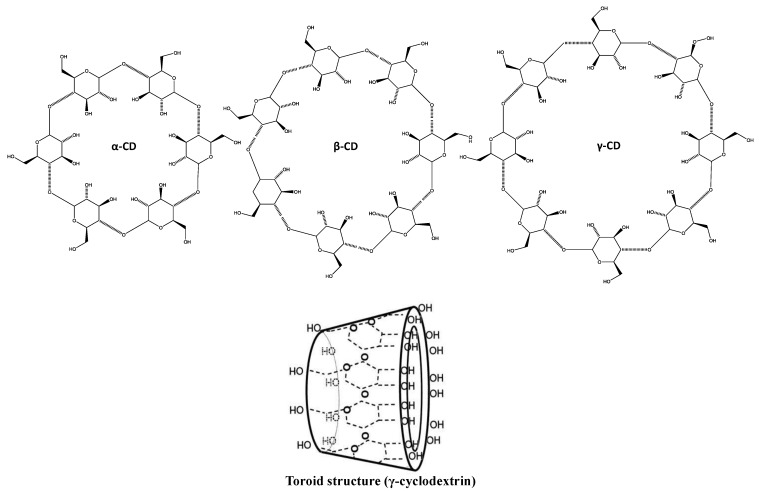
Chemical and toroid structure of cyclodextrins.

Cyclodextrins are a group of cyclic oligosaccharides that can be obtained from starch by enzymatic conversion. Two main topologies of native cyclodextrins can be found: crystalline and aqueous solution. Either channel-type and cage-type aggregates can be obtained in crystal structures [57]. Several parameters are known to influence the cyclodextrin size, including molar mass, concentration and host:guest molecule ratio [58]. Several derivatives exist, namely α-, β- and γ-cyclodextrin, which are 6, 7 and -8 membered, respectively (Figure 3).

There is a wide range of applications for cyclodextrins across several industries, namely pharmaceutical, food and cosmetic, mostly due to their low cost and toxicity. In addition, cyclodextrins are regarded as excellent options for masking odors and off-flavors [16].

Encapsulation involving cyclodextrins relies in selective molecular combination of host and guest molecules, ultimately yielding supramolecular systems [21].

Due to the chemical and physical properties of cyclodextrins, fatty acids and their methyl-, ethyl and saccharose esters, monoglycerides and similar long apolar alkane chain-bearing molecules, are regarded as good complex-forming guest molecules [17].

In what concerns to the use of cyclodextrins as carriers for ω-3 fatty acids, the authors report the influence of the β-cyclodextrins–fish oil ratio in several parameters. Among the several conditions tested, the ratio 10:20 was the one that scored best regarding encapsulation efficiency (84.1%), fish oil loading (62.7%), leakage after freeze-drying (11.0%) and EPA encapsulation efficiency (6.5%). After 3 days, freeze-dried β-cyclodextrins-fish oil retained 97% of fish oil within the particles [12]. The size of these particles was highly dependent on the fish oil loading, ranging from 250 to 700 nm, this last resulting from β-cyclodextrins:fish oil ratio of 10:20.

In another study cyclodextrins were used in combination with whey protein concentrate [59]. When evaluating several analogues of the cyclodextrins series, the γ- homologue scored better than β-cyclodextrin regarding emulsion stability and encapsulation efficiency. Whey protein concentrate:cyclodextrin ratio was the main factor affecting particle size, which ranged from 120 to 700 nm. Reduction of odor in the 70% range was also reported, as well as oxidative stability, as assessed by peroxide values [59].

#### 3.2.2. Caseins

**Figure 4:**
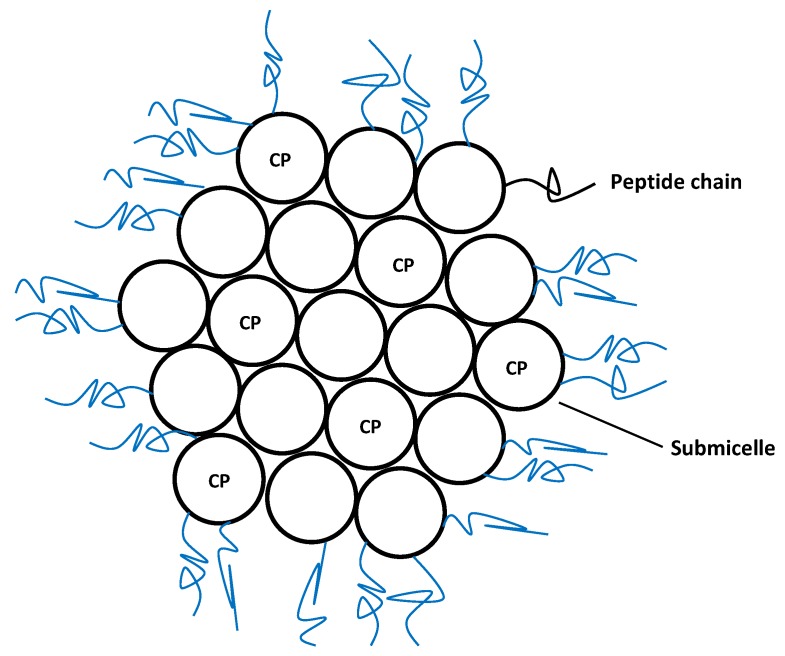
Structural arrangement of casein micelles. CP-Calcium phosphate.

Caseins are the major milk proteins, in which they occur in the form of large colloidal particles, casein micelles (Figure 4), presenting an average diameter of 150 nm. There are four main types of casein (αs1, αs2, β- and κ-casein), all of them being held together via hydrophobic and electrostatic interactions [38].

Experimentally, caseins are usually prepared by isoelectric precipitation of skim milk at pH lower than 5, with subsequent resolubilization with alkaline salts using calcium, potassium, sodium, or magnesium hydroxide to increase the pH to 6.7 and spray-drying to originate caseinates [60]. Structurally, the resulting micelles are composed of calcium phosphate clusters together with different casein molecules from the above mentioned families.

From a technological point of view, the most relevant characteristic of micelles, including caseins, is their ability to host non-polar molecules, hence improving their solubilization and increasing their bioavailability. For this reason, nowadays casein micelles are used for nano-encapsulation and stabilization of several components, notably in the food industry, where they are used to incorporate hydrophobic molecules in non-fat or low-fat products [38].

Caseins have been used to entrap and deliver DHA, one of the most important marine lipids. Each molecule of casein binds 3–4 DHA molecules, the resulting micelles displaying an average size of 50–60 nm when prepared at low temperatures in the presence of calcium and phosphate. Good colloidal stability and protective effect against DHA oxidation is observed at 4 °C [20].

In a different approach, caseinate was complexed with pectin in order to obtain biopolymer-based hydrogel microspheres, which were used to encapsulate fish oil [61]. This method has shown to improve the stability of fish oil concerning to lipid oxidation, as assessed by the low levels of hydroperoxides up to 7 days of storage. From a mechanistic point of view, this is believed to be a consequence of a high local concentration of antioxidant protein around the emulsified lipids [61].

#### 3.2.3. Zein Nanoparticles

Zein are a group of water-insoluble prolamine storage proteins that are mainly present in the endosperm of corn kernels [62]. Due to their organoleptic characteristics (odorless and tasteless), this material is a promising tool for carrying ingredients in delivery systems. Zein comprises at least four types of proteins, namely α-, β-, γ-, and δ-zein, each with different molecular weights and solubility. Industrially, commercial zein is obtained from corn gluten meal, a co-product of corn wet milling [63].

Several studies can be found addressing the use of zein as a carrier biopolymer for water-soluble molecules, such as heparin and ivermectin [64,65]. Concerning to marine fatty acids, fish oil has been encapsulated in zein particles in the nanometer range, namely 350–450 nm. Following freeze-drying, good oxidative stability was found for samples with zein-oil ratio of 4:1 or lower [66].

In another work using DHA instead of whole fish oil, Torres-Giner *et al*. used an ultrathin zein encapsulation approach [18]. This strategy revealed to increase the stability of DHA by reducing its degradation rate by around 2.5 fold and yielding substantially lower off-flavor content.

The digestion of DHA in fish oil encapsulated in porous starch granules coated with zein in the gastrointestinal tract of rats has also been addressed [19]. Initial *in vitro* studies showed that zein could be decomposed by proteolytic enzymes and *in vivo* experiments showed that particles loaded with fish/coconut/corn oil lowered serum triglycerides and phospholipids in rats. In the fish oil group the proportion of DHA in total fatty acids was significantly higher in both liver and brain. These results indicate that fish oil encapsulated in porous starch granules coated with zein are digested and absorbed [19].

## 4. Conclusions

An increasing number of works describe delivery systems that aim to increase the bioavailability of fatty acids and to protect them from oxidation, thus enhancing their absorption. In this regard, several systems, comprising distinct particle architectures, have been developed. While it is expected that in the next few years many advances in this field are likely to occur, some topics still require further attention.

In particular, it is important to address the *in vivo* bioavailability and pharmacokinetics of these systems, as many of the new micro- and nanoparticles have not yet been evaluated in this regard, only few works reaching the *in vivo* stage.

Despite the potential and academic interest of many of the systems discussed herein, when addressing this topic from an industrial perspective, some considerations should be taken into account. Regardless of the multiple advantages of carbohydrate and protein-based micro-/nanoparticles, they may hinder full scale-up due to technical requirement in terms of chemical or heat treatments. On the other hand, lipid-based particles may be easier to process at an industrial level and may pose advantages regarding encapsulation efficiency and toxicity.

Yet, another subject that requires attention is related with the lack of homogeneity regarding the parameters that are presented when characterizing micro/nanoparticles. For example, many works do not present data concerning to stability, the presence of oxidation products or encapsulation efficiency, which hinders the direct comparison of different techniques.
